# Vitamin E, Vitamin C, or Losartan Is Not Nephroprotectant against Cisplatin-Induced Nephrotoxicity in Presence of Estrogen in Ovariectomized Rat Model

**DOI:** 10.1155/2012/284896

**Published:** 2012-09-26

**Authors:** Mehdi Nematbakhsh, Zahra Pezeshki, Fatemeh Eshraghi-Jazi, Farzaneh Ashrafi, Hamid Nasri, Ardeshir Talebi, Tahereh Safari, Maryam Haghighi, Azam Mansouri

**Affiliations:** ^1^Water and Electrolytes Research Center, Isfahan University of Medical Sciences, HezarJerib Avenue, Isfahan 81745, Iran; ^2^Department of Physiology, Isfahan University of Medical Sciences, HezarJerib Avenue, Isfahan 81745, Iran; ^3^Kidney Diseases Research Center, Isfahan University of Medical Sciences, HezarJerib Avenue, Isfahan 81745, Iran; ^4^Department of Internal Medicine, Isfahan University of Medical Sciences, HezarJerib Avenue, Isfahan 81745, Iran; ^5^Department of Clinical Pathology, Isfahan University of Medical Sciences, HezarJerib Avenue, Isfahan 81745, Iran

## Abstract

*Background*. The nephroprotective effect of vitamins E and C or losartan against cisplatin (CP)- induced nephrotoxicity when they are accompanied by estrogen was investigated. *Methods*. The ovariectomized rats received estradiol valerate for two weeks. At the end of the first week, a single dose of CP (7 mg/kg, IP) was also administered, and they received placebo (group 1), vitamin E (group 2), vitamin C (group 3), or losartan (group 4) every day during the second week, and they were compared with another three control groups. *Results*. CP alone increased the serum levels of blood urea nitrogen (BUN), creatinine (Cr), and kidney tissue damage score (KTDS), significantly (*P* < 0.05), however at the presence of estradiol and CP, vitamin C, vitamin E, or losartan not only did not decrease these parameters, but also increased them significantly (*P* < 0.05). The serum level of superoxidase dismutase (SOD) was reduced by CP (*P* < 0.05), but it was increased when estradiol or estradiol plus vitamin C or losartan were added (*P* < 0.05). *Conclusion*. The particular pharmacological dose of estrogen used in this study abolish the nephroprotective effects vitamins C and E or losartan against CP-induced nephrotoxicity.

## 1. Introduction

Nephrotoxicity is the major adverse effect of cisplatin (CP) in about 30 percent of patients during chemotherapy in clinic due to tubular toxicity, inflammation, oxidative stress, and change in the renal circulation [1–4], and it is a limitation for CP use. To avoid this side effect and based on different experimental designs, protective role of administration of many synthetic or herbal agents as supplementation has been investigated against CP-induced nephrotoxicity [5–34], including vitamins E and C and losartan as antioxidant agents [5, 6, 22, 23]. During CP chemotherapy, plasma antioxidant concentrations decrease, and in human subject coadministrations of vitamin C, vitamin E, and selenium markedly increase plasma antioxidant levels [35]. In addition, the protective role of vitamin E supplementation against CP-induced peripheral neuropathy in patients was evaluated, and its neuroprotective role was reported [36, 37]. Although vitamins C and E and losartan have antioxidant effects [22, 23, 38, 39], losartan itself acts as a blocker of angiotensin II receptor 1 (ATR1), which facilitates renal circulation [40, 41]. The protective effect of losartan against CP-induced nephrotoxicity was reported before [5, 6], and it has no effect on CP uptake by the kidney [6]. However, acute administration of losartan does not affect the CP-induced kidney toxicity [5].

The protective role of estrogen in cardiovascular system in women before menopause is well documented [42–47]. The incidence of chronic renal diseases is also gender related and is potentially higher in males [48, 49], which may relate to estrogen-mediated vascular endothelial growth factor [48]. It is reported that estrogen receptors may be disturbed by CP, the steroid hormone increases the potency of CP [50], and estrogen may enhance oxidative stress in the kidney [51]. Therefore, not only the protective role of estrogen against CP-induced nephrotoxicity may be failed, but also its adverse side effect in CP-induced kidney toxicity became a question. Recently, we have compared the CP-induced kidney damage in male and female rats, and demonstrated that the CP-induced nephrotoxicity is gender related [52]. The protective role of losartan against CP-induced nephrotoxicity also indicates that losartan as an antioxidant is nephroprotectant in males but not in females [53]. So, one question is raised about the role of female sex hormone in this gender-based difference. Accordingly, we hypothesized that administration of estrogen and CP together promotes the potency of CP-induced nephrotoxicity, which abolishes the nephroprotective effects of antioxidants such as vitamins C and E and losartan. To test this hypothesis and in the first step, the effect of estrogen and its combination with vitamin E, vitamin C, or losartan in CP-induced nephrotoxicity should be determined. Therefore, ovariectomized rats were treated with the pharmacological dose of estradiol valerate. Then, particular doses of vitamin C, vitamin E, or losartan plus single dose of CP were administered, and the results were compared with those obtained from the control groups.

## 2. Materials and Methods

### 2.1. Animals

Forty-two adult female Wistar rats (Animal Centre, Ahvaz University of Medical Sciences, Ahvaz, Iran) with the mean weight of 182 ± 2.7 g were used. The rats were individually housed at a temperature of 23–25°C. Rats had free access to water and chow. The experimental procedures were in advance approved by the Isfahan University of Medical Sciences Ethics Committee.

### 2.2. Drugs

CP (cis-diammineplatinum (II) dichloride, code P4394), vitamin E, and vitamin C were purchased from Sigma (St. Louis, MO, USA). Estradiol valerate was obtained from Aburaihan Co. (Tehran, Iran). Losartan was provided by Darupakhsh Pharmaceutical Company (Tehran, Iran). 

### 2.3. Experimental Protocol

The animals were anesthetized by injection of 75 mg/kg, IP ketamine. An incision was made in the subabdominal region. The abdominal muscles were opened. The uterus tube and vascular base of ovaries were twisted, and the ovaries were removed. The muscles and skin were stitched backed into the place. After recovery, the animals were allowed to acclimatize to the same diet at least for one week. Then, they were randomly divided into seven experimental groups as follows.

Group 1: 2.5 mg/kg/week estradiol valerate in sesame oil was injected intramuscular for two weeks, and at the end of the first week, blood sample was obtained and a single dose of CP (7 mg/kg, IP) was also administered. Then, the animals received 0.5 mL/rat/day vehicle (saline) during the second week. 

Groups 2 to 4: the groups had regimen the same as group 1, except that vitamin E (1 g/kg/day, group 2), vitamin C (250 mg/kg/day, group 3), or losartan (10 mg/kg/day, group 4) was administered instead of vehicle during the second week. Blood samples were also obtained at the end of the first week.

Group 5 (positive control) received saline for two weeks, and at the end of the first week, blood sample was obtained. At the end of the first week, a single dose of CP alone was administered. Similarly, group 6 was treated by estradiol alone (2.5 mg/kg/week estradiol valerate for two weeks), and group 7 (negative control) received vehicle alone during the experiment.

At the end of the experiment (one week after CP injection), blood samples were obtained again and the rats were sacrificed by anaesthesia drug overdose. Kidneys and uterus were removed and weighted immediately, and the left kidney was prepared for histopathological procedures. Summary of the experimental protocol for each group of experiment is provided in Table 1.

### 2.4. Measurements

Body weight of the animals was recorded daily. The levels of serum creatinine (Cr), blood urea nitrogen (BUN), and magnesium (Mg) were determined using quantitative diagnostic kits (Pars Azmoon, Tehran, Iran). Serum level of nitrite (stable NO metabolite) was measured using a colorimetric ELISA kit (Promega Corporation, USA) that involves the Griess reaction. Serum level of estradiol was measured using enzyme immunoassay ELISA kit (Diagnostics Biochem Canada Inc., Canada). The serum level of superoxidase dismutase (SOD) was determined by a double-antibody sandwich enzyme-linked immunosorbent assay ELISA kits (Glory Science Co., USA). For each of the above-mentioned parameters, the concentration difference (Δ) was defined and calculated as (end of the second week (one week after CP administration) concentration − end of the first week (before CP administration) concentration).

### 2.5. Histopathological Procedures

The removed kidneys were fixed in 10% neutral formalin solution and embedded in paraffin for hematoxylin and eosin staining to examine the tubular damage. The damage was evaluated by two independent pathologists who were totally blind to the study. Based on the intensity of tubular lesions (hyaline cast, debris, vacuolization, flattening and degeneration of tubular cells, and dilatation of tubular lumen), kidney tissue damage score (KTDS) was graded from 1 to 4, while score zero was assigned to normal tubules without any damage. 

### 2.6. Statistical Analysis

Data are expressed as mean ± SEM. The percentage of changes in the body weight, the difference (after-before, Δ) of BUN, Cr, Mg, SOD, estradiol, and nitrite serum levels, and the kidney and the uterus weights in groups 1–4, 6, and 7 were compared with group 5 (the positive control group) using Student's *t*-test. The same comparison was applied to the KTDS using Mann-Whitney analysis. The *P* values ≤0.05 were considered statistically significant.

## 3. Results

The number of animals in each group and animals survived in the seven experiment groups are shown in Table 2. The mortality rate was more than 50 percent in groups 3 and 4 (Table 1). No mortality rate was observed in others groups.

### 3.1. Effect of CP: Comparison between Positive (Group 5) and Negative Control (Group 6 and 7) Groups

**Figure 1:**
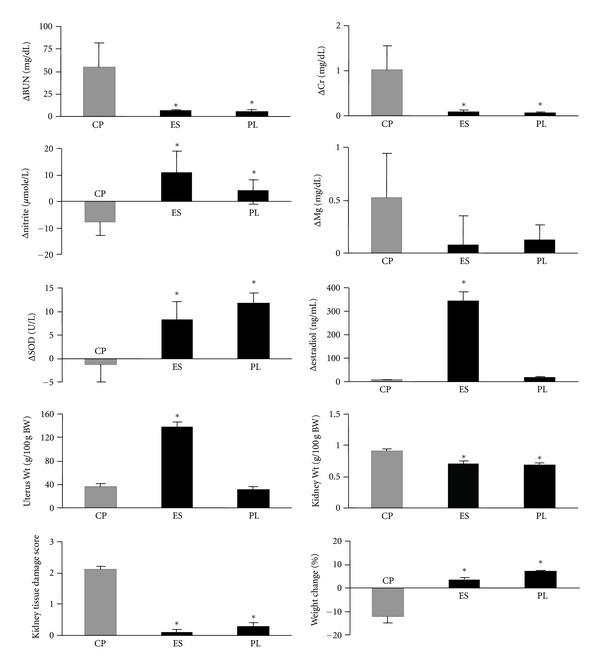
The serum difference levels (one week after CP administration − before CP administration; Δ) of BUN, Cr, Mg, SOD, estradiol, and nitrite; the kidney and the uterus weights; the KTDS in vehicle, estradiol, or CP (the positive control group) alone-treated ovariectomized groups. The star indicates significant difference (*P* < 0.05) when compared with the positive control groups. CP, ES, and PL on the horizontal axes stand for groups treated with CP (group 5), estradiol valerate (group 6), and vehicle (placebo, group 7), respectively.

The serum difference levels (one week after CP administration-before CP administration; Δ) of BUN, Cr, Mg, SOD, estradiol, and nitrite; the kidney and the uterus weights; the KTDS in vehicle alone, estradiol alone, or CP alone (the positive control group) treated ovariectomized groups are demonstrated in Figure 1. 

The serum levels of BUN and Cr, and KTDS significantly increased in the positive control group (*P* < 0.05) when compared with negative control groups (groups 6 and 7). This finding confirms kidney toxicity induced by CP. No significant differences were observed in the serum Mg levels between positive and negative control groups; indicating that no Mg depletion occurred by CP during one-week treatment with CP. Administration of CP reduced the serum levels of nitrite and SOD, while increase of these parameters was observed in the negative control groups (*P* < 0.05). The serum level of estradiol and uterus weigh in group 6 increased significantly (*P* < 0.05) when compared with groups 5 and 7; indicating that estradiol administration increases the blood estrogen level. 

### 3.2. Effect of Vitamin C, Vitamin E, or Losartan: Comparison between Positive Control (Group 5) and Case Groups (Groups 1, 2, 3, and 4)

**Figure 2:**
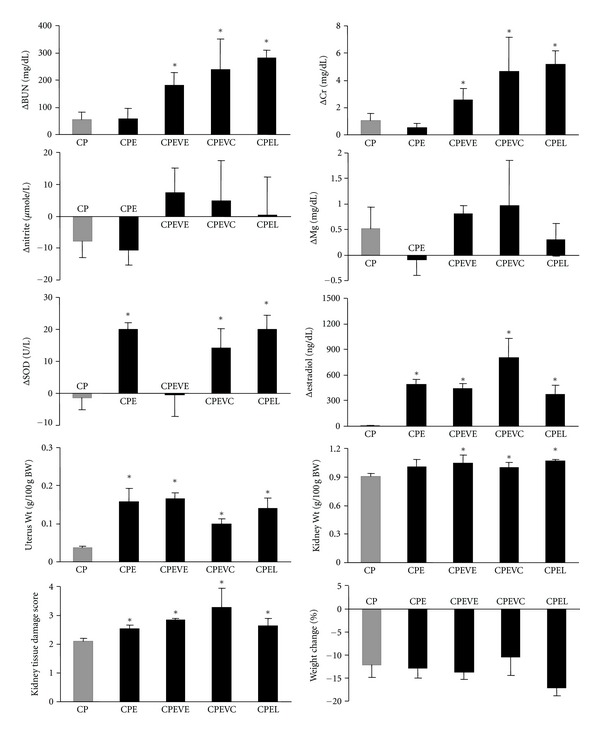
Serum difference levels (one week after CP administration − before CP administration; Δ) of BUN, Cr, Mg, SOD, estradiol, and nitrite; the kidney and the uterus weights; and KTDS in estradiol and CP, estradiol plus vitamin E and CP, estradiol plus vitamin C and CP, estradiol plus losartan and CP-treated ovariectomized groups (group 1–4) compared with ovariectomized rats treated with CP alone (positive control group). CP, CPE, CPEVE, CPEVC, and CPEL on the horizontal axes stand for groups treated with CP alone (group 5), estradiol and CP (group 1), estradiol plus vitamin E and CP (group 2), estradiol plus vitamin C and CP (group 3), and estradiol plus losartan and CP (group 4), respectively. The star indicates significant difference (*P* < 0.05) when compared with the positive control group.

The serum difference levels (one week after CP administration-before CP administration; Δ) of BUN, Cr, Mg, SOD, estradiol, and nitrite; the kidney and the uterus weights; KTDS in estradiol and CP (group 1), estradiol plus vitamin E and CP (group 2), estradiol plus vitamin C and CP (group 3), estradiol plus losartan and CP (group 4) treated ovariectomized groups in comparison with ovariectomized rats treated with CP alone (the positive control group; group 5) are demonstrated in Figure 2. At the presence of estradiol, vitamin C, vitamin E, or losartan not only did not decrease the serum levels of BUN and Cr, and kidney weight or KTDS in ovariectomized rats, but also significantly increased these parameters when compared with the positive control group (*P* < 0.05). No significant differences were observed in Δnitrite, ΔMg, and percentage change of weight between groups 1 to 5. The serum level of SOD in CP-treated rats was significantly increased by estradiol, estradiol plus vitamin C, and estradiol plus losartan (*P* < 0.05). Such finding was not observed for estradiol plus vitamin E. 

**Figure 3:**
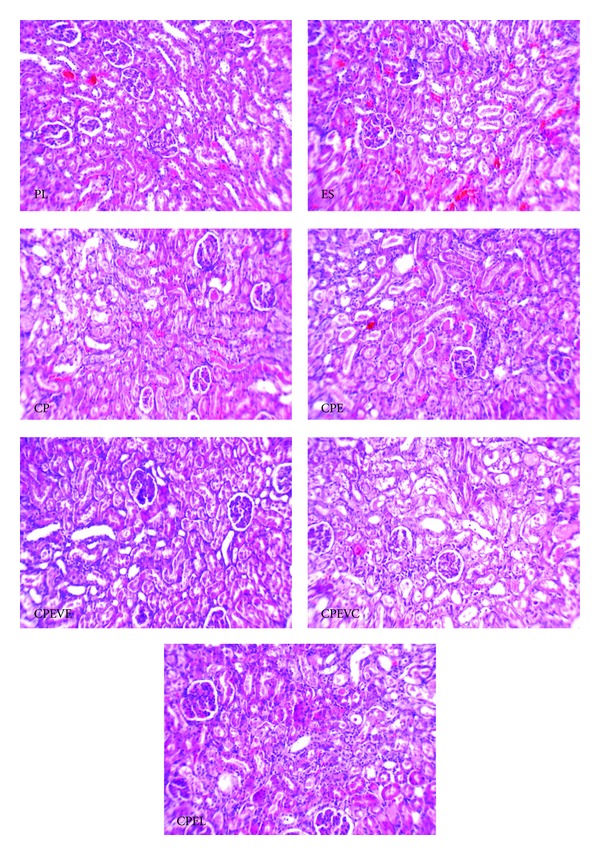
Images (magnification ×100) of kidney tissue. PL, ES, CP, CPE, CPEVE, CPEVC, and CPEL inside the images stand for groups treated with vehicle (placebo, group 7), estradiol valerate (group 6), CP alone (group 5), estradiol and CP (group 1), estradiol plus vitamin E and CP (group 2), estradiol plus vitamin C and CP (group 3), and estradiol plus losartan and CP (group 4), respectively. No tissue damages were seen in PL and ES, but higher and almost similar tissue damages were observed in other groups.

According to these findings, the vitamins or losartan has no beneficial effects against CP-induced nephrotoxicity when estradiol is present. Samples of kidney tissue images from each experimental group are shown in Figure 3.

## 4. Discussion

Our findings indicate that coadministration of vitamin C, vitamin E, or losartan with estradiol in female rats has no protective effect on the onset or severity of nephrotoxicity induced by CP, although previous reports indicated nephroprotectant effects for vitamins and ATR1 blocker, losartan, against CP-induced nephrotoxicity [5, 6, 22, 23]. The incidence rates of both cardiovascular and renal diseases are gender related and are higher in males. In the cardiovascular system, protective role of estrogen before menopause is well known [44–46]. However, estrogen may enhance oxidative stress in the kidney [51], and the oxidative stress induced by estrogen will enhance CP-induced injury to the tubules. In this study, we demonstrated for the first time that estrogen did not reduce the severity of nephrotoxicity, and actually it abolished the regular protective effect of antioxidants such as vitamin C, vitamin E, and losartan against CP-induced nephrotoxicity [34, 54–56], despite that estrogen itself has an antioxidant effect too. Instead of physiological dose of estrogen, we used the pharmacological dose of estradiol to establish the effect of estradiol first, and the result of this study strongly suggests examining the dose response of estradiol for future studies.

### 4.1. CP, BUN, Cr, Kidney Weight, Body Weight, Estrogen, and KTDS

According to the serum levels of BUN and Cr, and pathology damage score, nephrotoxicity was verified in all CP-treated animals (Figure 2). CP also caused weight reduction similar to our previous study [34]. The uterus weight increased in all estradiol-treated groups due to the presence of estradiol. The kidney tissue weight increases in CP-induced nephrotoxicity [57], and our findings were similar in this study. The KTDS in CP plus estradiol-treated groups either with or without vitamins or losartan was significantly greater than that in CP alone treated group. This data reveals the more significant role of coadministration of estrogen that abolishes the protective effect of vitamins or losartan against CP-induced nephrotoxicity. There are two possible explanations for the observation. Estrogen and CP may have two different effects on endothelial nitric oxide (NO). The first increases NO, and the second decreases the level [58–62]. These two opposite effects may restore the serum level of NO. However, estrogen-induced NO may enhance the severity of nephrotoxicity [62]. The second explanation is related to formation of oxidative stress induced by ovariectomy [63] and by CP administration [3]. These oxidative stresses play an effective role to promote the kidney injury. 

### 4.2. CP, Estrogen, and NO

CP alone causes dose-dependent declines in glomerular filtration rate (GFR) and renal blood flow due to renal vascular resistance changes [1, 4]. This phenomenon may limit CP transport to the kidney circulation. On the other hand, estrogen promotes formation of vasodilator biomarkers such as NO [58, 60, 61]. Our findings are in agreement with this item, as the serum level of NO metabolite (nitrite) elevated in the estrogen alone-treated group (Figure 1). NO involves in the process of CP-induced nephrotoxicity [64], and CP increases the level of inducible NO synthase [65] and decreases the level of endothelial NO synthase [59]. In addition, enhancement of NO level could increase the severity of toxicity [62]. It is also reported that inhibition of NO synthase aggravates CP-induced nephrotoxicity [57, 66]. Therefore, coadministration of CP and estrogen may promote the severity of toxicity due to estrogen-induced NO production. Another factor to consider is the availability of estrogen receptors in kidneys. Estradiol possibly sensitizes tubular cells to CP, similar to what happens in breast cancer cells [50], and promotes the tissue toxicity.

### 4.3. CP, Estrogen, and Oxidative Stress

It is well documented that abnormal production of reactive oxygen molecules, called oxidative stress, is involved in CP-induced nephrotoxicity, and CP produces renal oxidative stress, which disturbs renal function due to its toxicity [67]. Vitamins C and E reduce the level of SOD in CP-induced nephrotoxicity model [68]. Losartan also has antioxidant effect, and it may improve the renal function and decrease the severity of CP-induced nephrotoxicity [5, 6]. Estrogen also has antioxidant effect [54–56], and similar to vitamin E can modulate oxidative-stress-induced kidney toxicity [69, 70]. This is while other research indicated that estrogen enhances the oxidative stress in kidney [51]. In our study, the serum level of SOD was decreased by CP (Figure 1), and it was increased when estrogen alone or in combination of estrogen and vitamin C or losartan was administered. Opposite to these findings, Ulas and Cay [69] studied diabetic rat model, and no increment of SOD was detected in estradiol plus vitamin E treated rats. The level of serum SOD in ovariectomized rats treated either by estradiol or combination of estradiol and vitamin E was reported to be higher than nontreated ovariectomized animals [71], and this supplementation reduces lipid peroxidation [69]. But on the other hand, CP reduced the SOD level as an antioxidant defense system [72]. Considering our results, it seems that CP abolishes the antioxidant defense system induced by vitamin E and possibly estradiol due to possible pharmacokinetic or pharmacodynamic drug interactions. 

### 4.4. CP, Estrogen, and Mg

The serum levels of Mg in CP-treated groups were not statistically different. CP-induced nephrotoxicity disturbs tubular reabsorption of Mg, and depletion of Mg enhances nephrotoxicity [73–76]. The CP-induced hypomagnesemia is not related to the total dose of CP [77], and in animal model, hypomagnesemia develops from the third week after CP administration [78]. Furthermore, an increase in the serum Mg level after supplemental treatment is not expected [79], and the serum level of Mg did not change in ovariectomized rats treated with estradiol and vitamin E [71]. Accordingly, it seems that one week was not enough to observe Mg depletion by CP in our model.

## 5. Conclusion

In the presence of estradiol in the particular pharmacological dose used in the study, vitamins C and E or losartan could not be nephroprotectant against CP-induced nephrotoxicity. The exact mechanisms need to be defined, but it seems that coadministration of the agents with CP in ovariectomized rats promotes the oxidative stress and potency of CP, which contribute to nephrotoxicity severity. This contribution abolishes protective effect of the vitamins and losartan.

## Figures and Tables

**Table 1:** Summary of the treatment in the groups. Estradiol was injected at the beginning of each week. CP was administrated as a single dose at the beginning of the second week. The vitamins and losartan were injected daily during the second week.

Group	First week	Second week
1	Estradiol	Estradiol + CP + saline
2	Estradiol	Estradiol + CP + vitamin E
3	Estradiol	Estradiol + CP + vitamin C
4	Estradiol	Estradiol + CP + losartan
5	Saline	CP
6	Estradiol	Estradiol
7	Saline	Saline

**Table 2:** The mortality rate of animals in each group during the second week of the experiment.

Group	*N*	Day							*n*
		1	2	3	4	5	6	7	
Number 1: estradiol + CP	6	—	—	—	—	—	—	—	6
Number 2: estradiol + CP + vitamin E	7	—	—	—	—	—	—	—	7
Number 3: estradiol + CP + vitamin C	7	—	—	—	—	1	2	1	3
Number 4: estradiol + CP + losartan	7	—	—	—	—	1	1	2	3
Number 5: CP + vehicle (positive control)	5	—	—	—	—	—	—	—	5
Number 6: estradiol + vehicle (negative control)	5	—	—	—	—	—	—	—	5
Number 7: vehicle (negative control)	5	—	—	—	—	—	—	—	5

*N*: total number of animals at the beginning of experiment. All animals were ovariectomized, *n*: number of animals at the end of the experiment, CP: cisplatin. CP was administered at the beginning of the second week.
